# Liver-only metastatic colorectal cancer patients and thymidylate synthase polymorphisms for predicting response to 5-fluorouracil-based chemotherapy

**DOI:** 10.1038/sj.bjc.6604555

**Published:** 2008-08-12

**Authors:** F Graziano, A Ruzzo, F Loupakis, D Santini, V Catalano, E Canestrari, P Maltese, R Bisonni, L Fornaro, G Baldi, G Masi, A Falcone, G Tonini, P Giordani, P Alessandroni, L Giustini, B Vincenzi, M Magnani

**Affiliations:** 1 Medical Oncology, Azienda Ospedaliera ‘Ospedale San Salvatore’, Pesaro, Italy; 2 Institute of Biochemistry ‘G Fornaini’, University of Urbino, Urbino, Italy; 3 Medical Oncology, Hospital of Livorno, Livorno, Italy; 4 Medical Oncology, University Campus Biomedico, Rome, Italy; 5 Medical Oncology, Hospital of Fermo, Fermo, Italy; 6 Medical Oncology, Hospital of Livorno and University of Pisa, Pisa, Italy

**Keywords:** colorectal cancer, liver metastasis, thymidylate synthase, polymorphisms, 5-fluorouracil, pharmacogenetics

## Abstract

We investigated the association between thymidylate synthase (*TS*) germline polymorphisms and response to 5-fluorouracil-based chemotherapy in 80 patients with liver-only metastatic colorectal cancer (MCRC). The tandem repeat polymorphism (VNTR) in *TS* 5′-untranslated region (5′-UTR), which consists of two (*2R*) or three (*3R*) 28-bp repeated sequences, with or without a G/C nucleotide change in *3R* carriers (*3G* or *3C*) and a 6-bp insertion/deletion (*6*+/*6*−) in the *TS* 3′-UTR, was studied. The distinction between high (*2R/3G*, *3C/3G* and *3G/3G*) and low (*2R/2R*, *2R/3C* and *3C/3C*) *TS* expression genotypes according to the 5′-UTR VNTR+G/C nucleotide change showed significant association with tumour response (*P*=0.01). In particular, high TS expression genotypes were found in 8 out of 34 patients (23.5%) with complete or partial response and in 24 out of 46 patients (52%) with stable disease and disease progression. Liver-only MCRC patients are a homogeneous and clinical relevant subgroup that may represent an ideal setting for studying the actual influence of *TS* polymorphisms.

Functional polymorphisms in the 5′-untranslated region (5′-UTR) and the 3′-UTR of the thymidylate synthase (*TS*) gene have been identified in the last decade (Marsh, 2005). A variable number of 28-bp tandem repeated sequence (VNTR) in *TS* 5′-UTR determines two (*2R*) or three (*3R*) alleles (Horie *et al*, 1995) and three common genotypes (*2R/2R*, *2R/3R* and *3R/3R*). Upregulated TS protein levels were found to be associated with the *3R* allele (Kawakami *et al*, 1999; Yu *et al*, 2008). A *G/C* single-nucleotide polymorphism (SNP) in the *3R* allele was found to determine two additional alleles (*3G* or *3C*) at this locus (Kawakami and Watanabe, 2003; Mandola *et al*, 2003), and according to their functional role, it allows a distinction between high (*2R/3G*, *3C/3G* and *3G/3G*) and low (*2R/2R*, *2R/3C* and *3C/3C*) TS expression genotypes *in vivo* (Morganti *et al*, 2005; Yawata *et al*, 2005). A more recently discovered *TS* genetic variant is a 6-bp insertion/deletion (*6*+/*6*) in 3′-UTR (Ulrich *et al*, 2000). *TS* 3′-UTR genotypes (*6*+/*6*+−, *6*+/*6*− and *6*−/*6*−) seem to be associated with variable *TS* mRNA levels (Mandola *et al*, 2004); however, the functional effect of the 3′-UTR polymorphism is not well defined yet (Calascibetta *et al*, 2004).

5-Fluorouracil is a fundamental drug in the treatment of patients with colorectal cancer, and TS levels are considered an important factor for explaining the differences in 5-fluorouracil antitumour activity (Popat *et al*, 2004). Therefore, the *TS* functional polymorphisms are under investigation for the possibility of optimising chemotherapy (Yong and Innocenti, 2007). Studies in patients with metastatic colorectal cancer showed that carriers of the *TS 5′-UTR 3R* (*3G*) and/or the *TS 3′-UTR 6*+ alleles had adverse clinical outcomes (Pullarkat *et al*, 2001; Etienne *et al*, 2002; Park *et al*, 2002; Marcuello *et al*, 2004; Stoehlmacher *et al*, 2004; Martinez-Balibrea *et al*, 2007); however, such an association was not always detected (Lecomte *et al*, 2004; Jakobsen *et al*, 2005; Ruzzo *et al*, 2007a, 2007b). Heterogeneity in clinical experimental conditions (Sorbye *et al*, 2007), in tumour burden (Köhne *et al*, 2002) and in genetic/molecular features in the presence of a multisite metastatic disease (Yokota, 2000) may explain variable results in these pharmacogenetic studies.

We hypothesised that the 20–30% of patients with liver-only metastatic colorectal cancer (MCRC) (Mandalà *et al*, 2007) may represent a favourably homogeneous and clinically relevant setting for evaluating the role of *TS* polymorphisms for predicting response to 5-fluorouracil-based chemotherapy. For this purpose, we performed an analysis of *TS* polymorphisms in patients with liver-only MCRC who were previously enrolled in two prospective pharmacogenetic studies including 312 patients treated with first-line FOLFOX (bolus/infusional 5-fluorouracil coupled with oxaliplatin) or FOLFIRI (bolus/infusional 5-fluorouracil coupled with irinotecan) regimens (Ruzzo *et al*, 2007a, 2007b). FOLFOX and FOLFIRI regimens are equally active and they produce comparable response rates in first-line chemotherapy. In both the regimens, 5-fluorouracil is used at the same dose and schedule (Colucci *et al*, 2005). The primary end point of the study was the association between *TS* polymorphism and tumour response.

## Materials and methods

### Study population

Three hundred and twelve patients with metastatic colorectal cancer were prospectively enrolled in two previous pharmacogenetic studies (Ruzzo *et al*, 2007a, 2007b) and they underwent first-line chemotherapy including leucovorin 100 mg m^−2^ administered as a 2-h infusion before 5-fluorouracil 400 mg m^−2^ administered as an intravenous bolus injection and 5-fluorouracil 600 mg m^−2^ as a 22-h infusion immediately after FU bolus injection on days 1 and 2, every 2 weeks. Eighty patients (25.6%) had liver-only metastatic disease and they were included in this analysis. Ten of the 80 patients had history of liver surgery for metastasectomy and they were with liver-only relapse.

The 80 studied patients had cytologically or histologically confirmed metastatic colorectal cancer and the presence of at least one measurable lesion. Pretreatment evaluation included a complete medical and clinical–physical examination, KPS evaluation, baseline measurement of tumour size based on CT scan, serum chemistries and CEA. Objective response was evaluated after four cycles of treatment and then every 2 months according to the RECIST criteria (Therasse *et al*, 2000). For the purpose of this study, radiology studies of the 80 patients were reviewed for confirming the treatment outcomes. Patients’ characteristics and their outcomes were unknown to investigators performing genetic analyses. The study was approved by local ethical committees and patients provided signed informed consent.

### Analysis of polymorphisms

A blood sample from each enrolled patient was used for genotyping and it was collected before starting chemotherapy. Genomic DNA was extracted from 200 *μ*l whole blood using the QiaAmp kit (Qiagen, Valencia, CA, USA). All polymorphisms were investigated using a PCR-restriction fragment length polymorphism technique. The assays for studying polymorphisms were performed as described previously (Ruzzo *et al*, 2007a, 2007b).

### Statistical analyses

The primary end point of the study was the association between *TS* polymorphisms in patients with liver-only MCRC and response to 5-fluorouracil-based chemotherapy. Genotype frequencies were checked for agreement with those expected under the Hardy–Weinberg equilibrium. Genotypes for each polymorphism were analysed as three-group categorical variable in a codominant model and they were also grouped according to the recessive and additive model. Patients were categorised as responders (patients with complete or partial response) and non-responders (patients with stable disease or disease progression). The *χ*^2^-test was used for comparing proportions. Statistical significance was defined as *P*<0.05. A Bonferroni correction of the *P*-value for multiple comparisons was used where applicable.

The SHEsis software platform (http://202.120.7.14/analysis/myAnalysis.php) was used to estimate haplotype frequencies and the presence of linkage disequilibrium (LD). Linkage disequilibrium exists between two SNPs, if their variants appear together more often than expected (non-random inheritance). Linkage disequilibrium was estimated using *r*^2^, with *r*^2^=1 indicating complete LD and *r*^2^=0 indicating absent LD. Haplotype frequencies were reconstructed in the study population of responders and non-responders. Association of haplotypes with clinical outcome was estimated by comparing haplotype distributions among dichotomised patients using the *χ*^2^-test.

## Results

The characteristics of the 80 studied patients and the overall frequencies of genotypes are shown in Table 1. All patients were assessable for response and they received a minimum of four cycles of chemotherapy. In the 80 patients, there was one complete response (1.2%), 33 partial responses (41.2%), 30 stable diseases (37.6%) and 16 progressions (20%). Median age was 63 years (minimum 38 years and maximum 75 years). Liver metastases were synchronous in 22 patients (27.5%) and metachronous in 68 patients (72.5%). The frequencies of genotypes are in Hardy–Weinberg equilibrium and they are consistent with those observed in Caucasian ethnicity (Archive of Genetic Association Studies accessible at: http://geneticassociationdb.nih.gov/).

No significant association between clinicopathological features and tumour response was found (Table 2). The analysis of the three polymorphisms and response is shown in Table 3. The Bonferroni-adjusted *P*-value for the three comparisons is 0.05/3, *P*=0.016. The *TS* 5′-UTR VNTR with *G/C* polymorphism in *3R* alleles showed association with treatment outcome (*P*=0.011). In particular, high TS expression genotypes (*2R/3G*, *3C/3G* and *3G/3G*) were found in 8 out of 34 patients with complete or partial response (23.5%) and 24 out of 46 patients with stable disease and disease progression (52%). The 5′-UTR VNTR and the 3′-UTR 6-bp insertion/deletion (*6*+/*6*−) did not show association with tumour response. To further evaluate these two variants, their distribution was explored in recessive and additive models also, but without finding significant associations (Table 4).

The *TS* 5′-UTR and *TS* 3′-UTR loci showed mild LD (*r*^2^=0.17). As shown in Table 5, non-*3G* haplotypes were prevalent in responders and *3G* haplotypes in non-responders, with significantly different distribution of the *3G*/*6*− haplotype.

**Figure 1:**
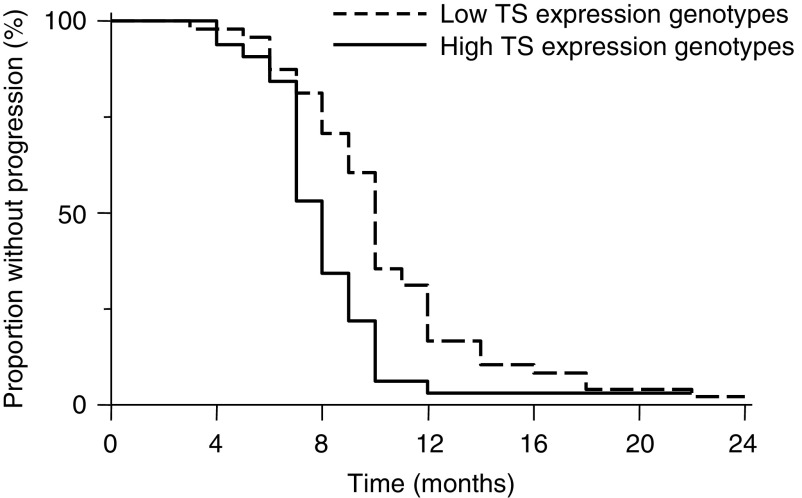
Kaplan–Meier analysis of time to progression (TTP) in patients with low *TS* expression genotypes (*2R/2R*, *2R/3C* and *3C/3C*) and high TS expression genotypes (*2R/3G*, *3C/3G* and *3G/3G*). TTP was calculated from the start of chemotherapy to the first evidence of disease progression. Two patients who underwent resection of liver metastases were progression-free at the time of analysis (one with a low TS expression genotype and the other with a high TS expression genotype). *χ*^2^ of the log-rank test=10.4 (*P*=0.001).

Eleven responsive patients underwent liver surgery for resection of the residual metastatic disease (13.7%). Clear resection margins with removal of all known metastatic lesions were attained in these patients, with 10 of them carrying one of the low TS expression genotypes (*2R/2R*, *2R/3C* and *3C/3C*). At the time of data analysis (March 2008), 78 patients suffered from disease progression (97.5%). For addressing an exploratory analysis of time to progression in patients with high and low *TS* expression genotypes, time to event distributions were studied using the Kaplan–Meier method. As shown in Figure 1, the results support the influence of the *TS* 5′-UTR VNTR with *G/C* SNP on the outcome of these patients.

## Discussion

To the best of our knowledge, this is the first analysis of *TS* polymorphisms in patients with liver-only MCRC. In comparison with previous studies (Pullarkat *et al*, 2001; Etienne *et al*, 2002; Park *et al*, 2002; Lecomte *et al*, 2004; Marcuello *et al*, 2004; Stoehlmacher *et al*, 2004; Jakobsen *et al*, 2005; Martinez-Balibrea *et al*, 2007; Ruzzo *et al*, 2007a, 2007b), we evaluated a homogeneous and low-burden disease population that was exposed to the same regimen of 5-fluorouracil including both bolus and infusional administration of the drug (Hoshino *et al*, 2005).

Heterogeneity in clinical features in patients with metastatic disease (Sorbye *et al*, 2007) may represent a major limitation for observing actual pharmacogenetic effects of functional germline polymorphisms. Notably, the number of metastatic sites plays a relevant prognostic role in patients with metastatic colorectal cancer (Köhne *et al*, 2002), and differences in clinical outcomes can be observed between patients with different metastatic sites (Köhne *et al*, 2002). The planning of this study was also motivated by the fact that colorectal cancer commonly metastasises to the liver, and such a single-organ involvement, instead of a multisite metastatic disease, can accurately be monitored during chemotherapy for the assessment of response (Trillet-Lenoir *et al*, 2002). Low-burden metastatic disease may also limit the impact of heterogeneity in molecular/genetic alterations as they accumulate during tumour progression and metastatisation (Yokota, 2000). Loss of heterozygosity (LOH) is to be included among these possible genetic changes. Owing to LOH at the *TS* locus on chromosome 18 in cancer cells, carriers of the germline heterozygous *2R/3R* genotype can acquire the *2R/loss* genotype in their tumours (Kawakami *et al*, 2002; Uchida *et al*, 2004a, 2004b). Therefore, the lower responsiveness of germline *2R/3R* carriers could not be displayed because heterozygous patients with tumour *2R/loss* genotype behave as *2R/2R* patients (Uchida *et al*, 2004a, 2004b). This phenomenon implies that responsiveness to 5-fluorouracil-based therapy may depend on the tumour rather than on the germline status of the genotypes (Uchida *et al*, 2004a, 2004b). However, our findings suggest that the double assessment of the VNTR plus G/C nucleotide change with dichotomisation of patients into carriers of high (*2R/3G*, *3C/3G* and *3G/3G*) and low (*2R/2R*, *2R/3C* and *3C/3C*) TS expression genotypes may not suffer from the possible presence of LOH. The finding of the association between *TS* 5′-UTR VNTR+G/C and tumour response may not only reflect a better functional characterisation of *3C* and *3G* alleles, but also a less extensive influence of tumour LOH on the germline assessment for high-TS expression genotypes (*2R/3G*, *3C/3G* and *3G/3G*).

In our opinion, *TS* polymorphisms deserved the present investigation more than other genetic variants with putative influence on 5-fluorouracil activity (i.e. methylenetetrahydrofolate reductase gene polymorphisms). In addition to the fact that TS is the target enzyme of 5-fluorouracil, it has been observed that TS levels may be dynamic, with upregulation after fluoropyrimidine exposure (Uchida *et al*, 2004a, 2004b; Mauritz *et al*, 2007). In particular, this effect was described in liver metastases from colorectal cancer in patients who received bolus 5-fluorouracil (Mauritz *et al*, 2007). Therefore, *TS* polymorphisms may influence the outcome to 5-fluorouracil chemotherapy, not only for their role in determining different baseline levels of TS activity (Marsh, 2005), but also for modulating the enhancement of TS levels in response to 5-fluorouracil. In fact, the 5-fluorouracil-induced upregulation of *TS* mRNA may be greater in carriers of high-expression *TS* genotypes than in carriers of low-expression *TS* genotypes. We cannot rule out, however, that a double assessment of *TS* and methylenetetrahydrofolate reductase polymorphisms may improve the predictive role of the single analysis of *TS* polymorphisms.

Another reason for studying pharmacogenetics in liver-only MCRC patients is related to the lack of predictive factor for response to neoadjuvant chemotherapy. Liver surgery can provide long-term survival for liver-only, metastatic colorectal cancer patients, but liver metastasectomy is feasible in only 15–25% of the patients. Neoadjuvant chemotherapy can provide response rates as high as 50%, allowing liver metastasectomy in about 10–15% of patients initially deemed unresectable. Tumour response to preoperative chemotherapy seems to be associated with outcome following liver resection for colorectal metastases (Folprecht *et al*, 2005) and, if genetically predictable, it could be improved by the selective choice of available drugs. In this study, 10 of the 11 responsive patients who underwent liver surgery were carriers of low TS expression genotypes (*2R/2R*, *2R/3C* and *3C/3C*). Actually, we performed an analysis of *TS* polymorphisms for response to 5-fluorouracil and we did not address this study to pharmacogenetics for liver metastases resectability and survival after preoperative chemotherapy. These end points require a prospective study, including a baseline multidisciplinary evaluation of the unresectable liver disease and long-term follow-up.

In conclusion, the homogeneous subgroup of patients with liver-only metastatic disease allowed the predictive role of *TS* polymorphisms to stand out. In fact, the association between polymorphisms and tumour response was included in the secondary end points of our two previous pharmacogenetic studies (Ruzzo *et al*, 2007a, 2007b), but these analyses failed to demonstrate a predictive role for the genetic variants. The clinical setting assumes a relevant role for exploring the pharmacogenetic associations in patients with metastatic cancer and additional studies are warranted for confirming our findings.

## Figures and Tables

**Table 1:** Characteristics of the 80 patients and genotype frequencies

	**No. of patients (%)**
*Sex*	
Male	48 (60)
Female	32 (40)
	
*Karnofsky performance status*	
90–100	58 (72)
80	22 (28)
	
*Resected primary tumour*	
Yes	70 (87)
No	10 (13)
	
*Prior adjuvant therapy*	
None	44 (55)
Yes	36 (45)
	
*Carcinoembryonic antigen*	
⩽10 ng ml^−1^	56 (70)
>10 ng ml^−1^	24 (30)
	
*Genotypes*	
*TS* 5′-*UTR VNTR*^a^	
*2R/2R*	16 (20)
*2R/3R*	38 (47)
*3R/3R*	26 (33)
	
*TS 5′-UTR VNTR*+*G/C*^b^	
*2R/2R*, *2R/3C*, *3C/3C*	48 (60)
*2R/3G*, *3C/3G*, *3G/3G*	32 (40)
	
*TS 3′-UTR*	
−*6*/−*6*	30 (38)
−*6*/+*6*	40 (50)
+*6*/+*6*	10 (12)

a The variable number of tandem repeats (VNTR) polymorphism is a two (*2R*) or three (*3R*) 28-bp tandem repeat sequence in *TS* 5′-UTR.

b A single-nucleotide change in *3R* allele is a second polymorphism that distinguishes *3G* carriers (*2R/3G*, *3G/3G* and *3G/3C* genotypes) from non-*3G* carriers (*2R/2R*, *2R/3C* and *3C/3C* genotypes).

**Table 2:** Characteristics of the patients and tumour response

		**No. of patients (%)**		
**Characteristics**	**No. of patients (%)**	**Responders^a^ (*N*=34)**	**Non-responders^a^ (*N*=46)**	***P*-value**
*Karnofsky performance status*				
90–100	58 (72)	26 (76)	32 (70)	0.6
80	22 (28)	8 (24)	14 (30)	
				
*Resected primary tumour*				
Yes	67 (84)	30 (88)	37 (80)	0.5
No	13 (16)	4 (12)	9 (20)	
				
*Prior adjuvant therapy*				
None	44 (55)	21 (62)	23 (50)	0.4
Yes	36 (45)	13 (38)	23 (50)	
				
*Carcinoembryonic antigen*				
⩽10 ng ml^−1^	56 (70)	26 (76)	30 (65)	0.4
>10 ng ml^−1^	24 (30)	8 (24)	16 (35)	

a Responders are patients with complete or partial response. Non-responders are patients with stable disease or disease progression.

**Table 3:** Association between genotypes and response to chemotherapy in the 80 patients

		**No. of patients (%)**		
**Genotypes**	**No. of patients (%)**	**Responders^a^ (*N*=34)**	**Non-responders^a^ (*N*=46)**	***P*-value**
*TS 5′-UTR*				
*2R/2R*	16 (20)	10 (30)	6 (13)	
*2R/3R*	38 (47)	14 (40)	24 (52)	0.19
*3R/3R*	26 (33)	10 (30)	16 (35)	
				
*TS 5′-UTR* b				
*2R/2R*, *2R/3C*, *3C/3C*	48 (60)	26 (76)	22 (48)	0.011§
*2R/3G*, *3C/3G*, *3G/3G*	32 (40)	8 (24)	24 (52)	
				
*TS 3′-UTR*				
−*6*/−*6*	30 (38)	11 (32)	19 (41)	
−*6*/+*6*	40 (50)	20 (59)	20 (44)	0.37
+*6*/+*6*	10 (12)	3 (9)	7 (15)	

a Responders are patients with complete or partial response. Non-Responders are patients with stable disease or disease progression.

b Analysis of the *TS* 5′-UTR VNTR with *C/G* nucleotide change in *3R* allele carriers. Low-expression genotypes are *2R/2R*, *2R/3C* and *3C/3C*. High-expression genotypes are *2R/3G*, *3G/3G* and *3G/3C*.

§ *P*<0.016 is the level of significance according to the Bonferroni adjustment for three comparisons.

**Table 4:** Association between genotypes and response to chemotherapy in additive and recessive models

		**No. of patients (%)**		
**Genotypes**	**No. of patients (%)**	**Responders^a^ (*N*=34)**	**Non-responders^a^ (*N*=46)**	***P*-value**
*TS 5′-UTR*				
*Recessive model*				
*2R/2R*, *2R/3R*	54 (67)	24 (70)	30 (65)	0.63
*3R/3R*	26 (33)	10 (30)	16 (35)	
				
*TS 5′-UTR*				
*Additive model*				
*2R/2R*	16 (20)	10 (30)	6 (13)	0.09
*2R/3R*, *3R/3R*	64 (80)	24 (70)	40 (87)	
				
*TS 3′-UTR*				
*Recessive model*				
−*6*/−*6*, −*6*/+*6*	70 (87)	31 (91)	39 (85)	0.50
+*6*/+*6*	10 (13)	3 (9)	7 (15)	
				
*TS 3′-UTR*				
*Additive model*				
−*6*/−*6*	30 (38)	11 (32)	19 (41)	0.48
−*6*/+*6*, +*6*+*6*	50 (62)	23 (68)	27 (59)	

a Responders are patients with complete or partial response. Non-responders are patients with stable disease or disease progression.

**Table 5:** Distribution of estimated haplotype frequencies according to treatment outcome

**Haplotype**	**Non-responders (%)**	**Responders (%)**	***χ* ^2^**
*2R/6*−	7.3	9.7	0.3
*2R/6*+	32.5	41.3	0.06
*3C/6*−	8.9	9.6	0.7
*3C/6*+	16.3	19.9	0.2
*3G/6*−	26.2	16.9	0.01
*3G/6*+	6.2	5.2	0.5
